# Integrative Lipidomics and Metabolomics for System-Level Understanding of the Metabolic Syndrome in Long-Term Treated HIV-Infected Individuals

**DOI:** 10.3389/fimmu.2021.742736

**Published:** 2022-01-12

**Authors:** Sofie Olund Villumsen, Rui Benfeitas, Andreas Dehlbæk Knudsen, Marco Gelpi, Julie Høgh, Magda Teresa Thomsen, Daniel Murray, Henrik Ullum, Ujjwal Neogi, Susanne Dam Nielsen

**Affiliations:** ^1^ Department of Infectious Diseases, Copenhagen University Hospital Rigshospitalet, Copenhagen, Denmark; ^2^ National Bioinformatics Infrastructure Sweden (NBIS), Science for Life Laboratory, Department of Biochemistry and Biophysics, Stockholm University, Stockholm, Sweden; ^3^ Personalized Medicine of Infectious Complications in Immune Deficiency (PERSIMUNE), Rigshospitalet, Copenhagen, Denmark; ^4^ Department of Clinical Immunology, Copenhagen University Hospital, Copenhagen, Denmark; ^5^ The Systems Virology Lab, Division of Clinical Microbiology, Department of Laboratory Medicine, Karolinska Institute, ANA Futura, Stockholm, Sweden; ^6^ Manipal Institute of Virology (MIV), Manipal Academy of Higher Education, Manipal, India

**Keywords:** HIV-1, metabolic syndrome, antiretroviral treatment, machine learning, lipidomics

## Abstract

People living with HIV (PLWH) require life-long anti-retroviral treatment and often present with comorbidities such as metabolic syndrome (MetS). Systematic lipidomic characterization and its association with the metabolism are currently missing. We included 100 PLWH with MetS and 100 without MetS from the Copenhagen Comorbidity in HIV Infection (COCOMO) cohort to examine whether and how lipidome profiles are associated with MetS in PLWH. We combined several standard biostatistical, machine learning, and network analysis techniques to investigate the lipidome systematically and comprehensively and its association with clinical parameters. Additionally, we generated weighted lipid-metabolite networks to understand the relationship between lipidomic profiles with those metabolites associated with MetS in PLWH. The lipidomic dataset consisted of 917 lipid species including 602 glycerolipids, 228 glycerophospholipids, 61 sphingolipids, and 26 steroids. With a consensus approach using four different statistical and machine learning methods, we observed 13 differentially abundant lipids between PLWH without MetS and PLWH with MetS, which mainly belongs to diacylglyceride (DAG, n = 2) and triacylglyceride (TAG, n = 11). The comprehensive network integration of the lipidomics and metabolomics data suggested interactions between specific glycerolipids’ structural composition patterns and key metabolites involved in glutamate metabolism. Further integration of the clinical data with metabolomics and lipidomics resulted in the association of visceral adipose tissue (VAT) and exposure to earlier generations of antiretroviral therapy (ART). Our integrative omics data indicated disruption of glutamate and fatty acid metabolism, suggesting their involvement in the pathogenesis of PLWH with MetS. Alterations in the lipid homeostasis and glutaminolysis need clinical interventions to prevent accelerated aging in PLWH with MetS.

## Introduction

Combination antiretroviral therapy (cART) has increased life expectancy for people living with HIV (PLWH). However, an increase in incidences of comorbidities such as obesity, type 2 diabetes (T2D), and cardiovascular disease (CVD) related to metabolic syndrome (MetS) (i.e., abdominal obesity, hypertension, elevated levels of triglycerides, dyslipidemia, and altered glucose levels), has become a growing concern in successfully treated PLWH. In chronic HIV infection, complicated interactions between effects of persistent low-grade immune activation, metabolic toxicity from cART, and non-HIV-related risk factors may increase the risk of MetS in PLWH. However, the pathophysiology of MetS in PLWH is still incompletely understood (1–3).

cART is known to be associated with changes in fat distribution (i.e., lipodystrophy and dyslipidemia) and metabolic abnormalities due to side effects (4). A few studies have investigated the association of HIV infection and cART with metabolic abnormalities related to MetS (4, 5). These studies have focused on conventional blood lipids, such as triglyceride and total cholesterol. These biomarkers may not sufficiently reflect the complex alterations of the lipid metabolism in PLWH with MetS.

Plasma lipidomics studies in the general population have identified several lipid species within the lipidome to be associated with features of MetS (6). In particular, HIV infection has been recently described by our group to be independently associated with a 1.5 fold increased risk of MetS compared to the general population (5). In addition, obesity has been shown to increase the content of almost all detectable diacylglyceride (DAG) and triacylglyceride (TAG) lipid species, along with several cholesteryl esters (CE), phosphatidylcholine (PC), phosphatidylethanolamine (PE), and lysophosphatidylcholine (LPC) in a general population (7). The pathophysiology and alterations of the lipidome in PLWH with MetS are yet to be explored and may help in the discovery of new patterns and disease markers associated with MetS in PLWH (3). In prior work from our group, we identified key metabolites, which influenced and altered the metabolome of PLWH with MetS (8).

In this study, an exploratory analysis of the lipidome comparing PLWH without MetS and PLWH with MetS was conducted to identify a set of key lipids that define the mechanism of the lipid abnormalities of MetS in the context of HIV infection. Also, we have performed advanced network analysis that revealed deeper underlying patterns within the metabolome (i.e., the polar metabolome and lipidome) of PLWH with MetS. Additionally, we investigated the influence of clinical demographic parameters on integrative metabolomics and lipidomics to provide snapshots of the biological phenotypes linked with MetS in PLWH.

## Materials and Methods

### Study Designing, and Patients

We obtained data from the Copenhagen Comorbidity in HIV Infection (COCOMO) study (9), an ongoing non-interventional, observational, longitudinal cohort study to assess the burden of non-AIDS comorbidities in PLWH. Sample collections and quantifications of the COCOMO cohort have previously been described (5, 9). Of the 1099 participants in the COCOMO study, 100 PLWH ≥ 40 years old were included and matched according to age, sex, duration of cART, smoking status, and current CD4+ T-cells count to 100 PLWH without MetS (5, 9). The MetS was defined according to the International Diabetes Federation (IDF) consensus worldwide definition of the MetS as previously (8, 10). For each individual, we collected clinical data from the COCOMO database with the following 13 HIV and MetS specific variables. The MetS, sex, age, ethnicity, immunodeficiency (i.e., lowest CD4+ T-cell count < 200 cells/*µ*l or previous AIDS condition), exposure to early-generation antiretroviral therapy (ART) (i.e., medicated with thymidine analogs, didanosine and/or indinavir), visceral adipose tissue (VAT) [cm2], subcutaneous adipose tissue (SAT) [cm2], and ART drugs including the active agents; nucleotide reverse transcriptase inhibitors (NRTIs), non-nucleotide reverse transcriptase inhibitors (NNRTIs), protease inhibitors (PIs), integrase strand transfer inhibitors (INSTIs), and other/unknown active agents). Furthermore, a lipidomics dataset (see below) and a metabolomics dataset with 11 key metabolites [i.e., 1-carboxyethylisoleucine, 4-cholesten-3-one, 4-hydroxyglutamate, *α*-ketoglutarate, carotene diol(2), *γ*-glutamylglutamate, glutamate, glycerate, isoleucine, pimeloylcarnitine/3-methyladipoylcarnitine (C7-DC) (PC/3-MAPC), and palmitoyl-sphingosinephosphoethanolamine (d18:1/16:0) (PSP)] previously identified by using a combination of standard biostatistical, machine learning and network analysis technique, were collected (8). Ethical approval was obtained by the Regional Ethics Committee of Copenhagen (COCOMO: H-15017350). Written informed consent was obtained from all participants.

### Plasma Lipidomic Profiling

Untargeted lipidomic profiling was performed on plasma samples collected at baseline in COCOMO through the Complex Lipid Panel*™* technique (Metabolon Inc, Morrisville, NC 27560, USA). The company is ISO 9001: 2015 certified for analytical and diagnostic testing of biological specimens. Briefly, lipids were extracted from the bio-fluid using automated BUME extraction followed by infusion-MS analysis (11). Lipid species were quantified by taking the ratio of the signal intensity of each target compound to that of its assigned internal standard, then multiplying by the concentration of internal standard added to the sample. Lipid class concentrations were calculated, and fatty acid (FA) compositions were determined by calculating the proportion of each class comprised by summation of individual FAs. All the lipid quantifications were median-centered, and missing values were minimum-imputed per lipid species. We further removed variables with zero or near-zero variance from the dataset using *nearZeroVar* (i.e., 5%, n = 46 of 963). The original scale lipidomics data can be obtained from **Supplementary Data File S1**.

### Statistics and Bioinformatics Analysis

All the analyses were carried out in R 4.0.3 (12). Clinical characteristics between PLWH without MetS and PLWH with MetS were compared using the Mann–Whitney U test (continuous variables) and chi-square test (categorical variables). Dimension reduction was carried out using principal component analysis (PCA). Structural interpretation of the lipidome was carried out through *lipidomeR.* The normality of the lipidomics data was tested through Kolmogorov-Smirnov test and density plots (13). The Mann-Whitney U test was applied to raw data and a subset of lipids with a false discovery rate (FDR)<0.001, was derived. Log-transformed data were tested for differential abundance using *limma* and significant lipids with a FDR<0.001, were derived (14). Binary classification modeling was carried out by partial least squares discriminant analysis (PLS-DA) using *ropls* (15), where a subset of variables with variables importance on projection (VIP) score >1 was derived. Random forest (RF) was carried out using *MUVR* [https://github.com/CarlBrunius/MUVR]. Variables from the optimal RF modeling performance were selected according to rank. Model performance was evaluated by using the Q2Y and area under the receiver operating characteristic (AUROC) for PLS-DA and RF, respectively. Pathway enrichment was tested from the *limma* output (FDR < 0.1) with Ingenuity Pathway Analysis (IPA) (Qiagen, US) and MetaboAnalyst (16) (limma, FDR< 0.1). The FDR was controlled for by using the Benjamin-Hochberg (BH) method (13).

### Network Analysis

Network analyses were used to build a biological network consisting of lipids (n = 917) and previously identified key metabolites (n = 11) (8) after Spearman’s rank correlation across all species. Edges connecting nodes (i.e., biomolecules) were weighted based on positive correlations. This network was compared against a null model attained from a random network with the same number of nodes and edges based on the Erdos-Renyi model (17). All networks were built through the Python module *igraph* (18), communities within the biological network were detected through the Leiden algorithm (19). Communities were characterized functionally and phenotypically through the lipid-specific ontology web tool, LION/web (20). LION/web was used to determine lipid ontology trends within each community, using all lipids from the network as a background list. Separate analyses on each network community with all lipids as background lists were uploaded to LIPEA to identify lipid pathway enrichment (21). The community association with clinical parameters was determined through logistic and linear regression in R. Network visualization was performed using Cytoscape 3.5.1 (22).

## Results

### Machine Learning Highlights Differences in Key Lipids in PLWH With MetS

**Figure 1:**
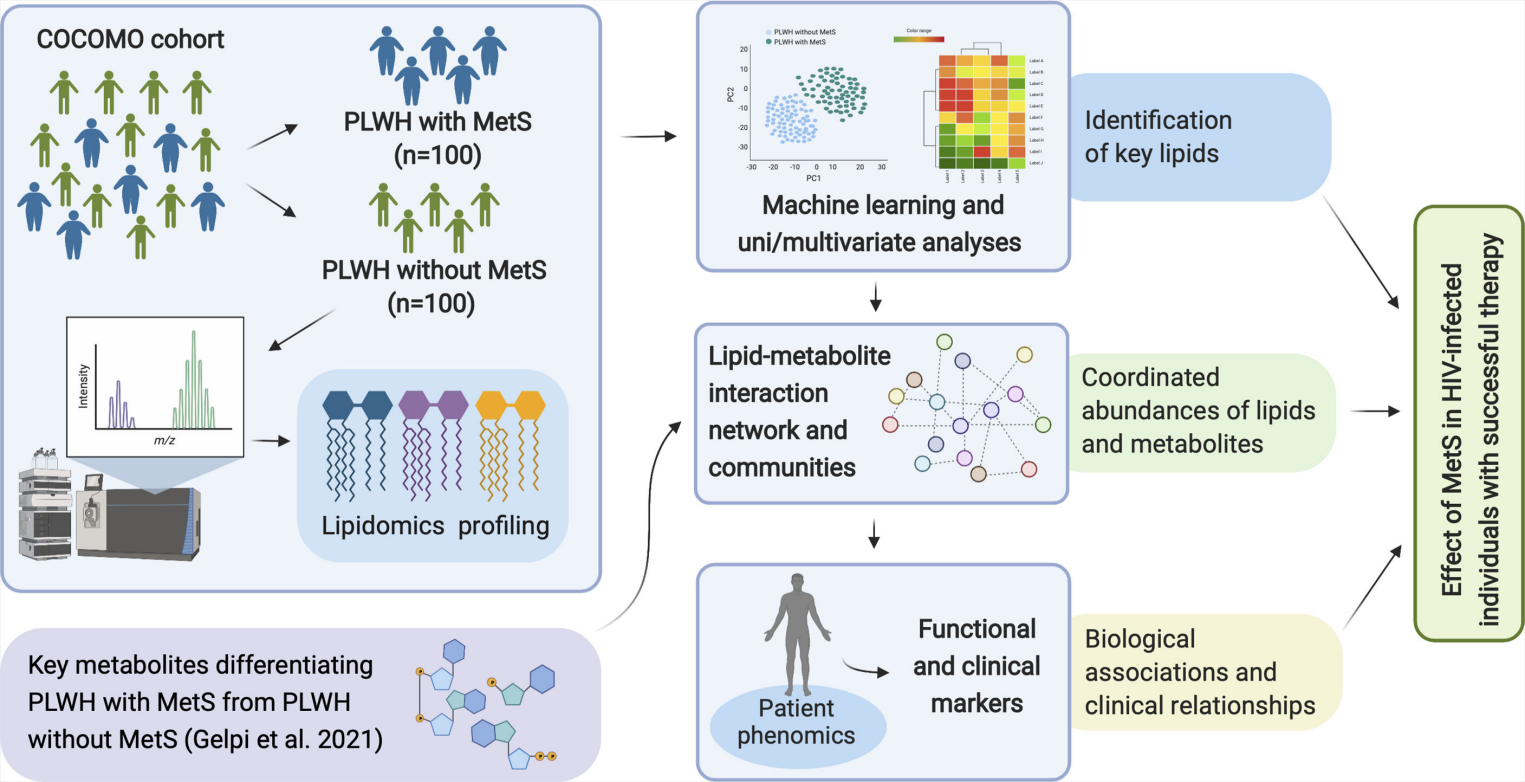
Overview of study workflow. Analysis pipeline for characterizing the effect of MetS in HIV-infected following ART treatment and investigating the underlying biological mechanisms of PLWH with MetS (created with BioRender.com).

**Figure 2:**
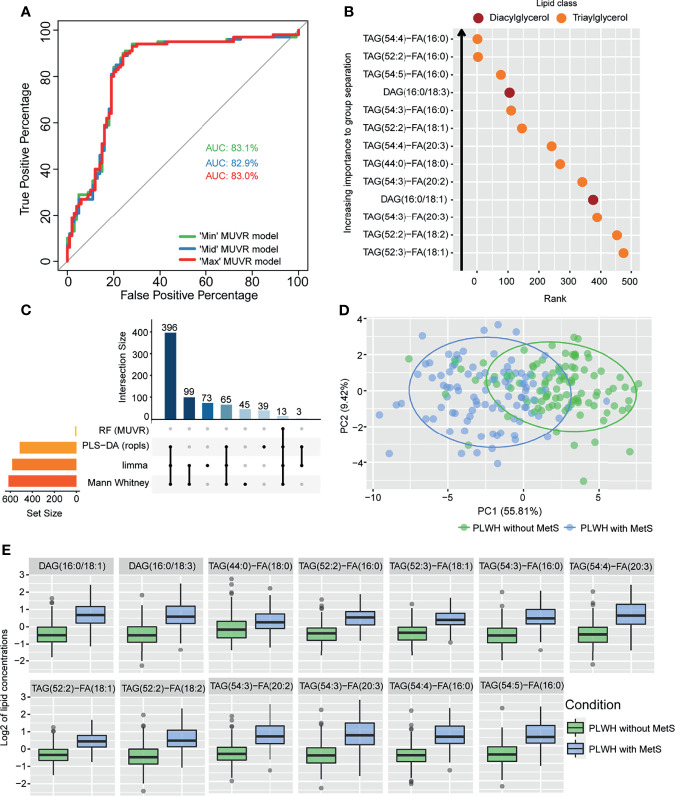
Lipidomics analyses of PLWH without MetS vs PLWH with MetS identifying key lipids differentiating the two groups. **(A)** Performance of random forest (RF) models. Receiver operating characteristic (ROC) curve with area under the curve (AUC) values for the three *MUVR* models. **(B)** Important prediction variables separating PLWH without MetS from PLWH with MetS based on lipidomics, diacylglycerol (DAG) and triacylglycerol (TAG). Variable’s importance on projection (VIP) scores plot for the ‘Max’ *MUVR* model, where lower rank indicates better group separation, thus better prediction variables in the model classification. **(C)** The intersection of methods identifying key lipids. UpSet plot showing number of significant lipids found *via* four statistical methods (RF, PLS-DA, limma, and Mann-Whitney U test). Note the 13 lipids (intersection size on the y-axis) are simultaneously identified by all four methods. **(D)** Separation of PLWH without MetS from PLWH with MetS based on identified key biomolecules. Principal component analysis (PCA) on key biomolecules, where lipidomics and metabolomics data were separated by the 13 identified key lipids and 11 identified key metabolites (**Table 2**). Ellipses show the 95% confidence interval of the data. **(E)** Boxplot of lipid concentration of the identified key lipids, which consist of DAGs and TAGs.

PLWH with MetS (n = 100) and PLWH without MetS (n = 100) were included from the COCOMO study (**Table 1**). VAT and SAT significantly differed between the two groups (p-value<0.001). We further applied several univariate and machine learning approaches to characterize the effect of MetS in HIV-infected following long-term cART treatment and to investigate the underlying biological mechanisms of MetS (**Figure 1**). The lipidomic dataset consisted of 917 unique lipid species including 602 glycerolipids, 228 glycerophospholipids, 61 sphingolipids, and 26 steroids. We observed 618 and 584 significantly differentially abundant lipids between PLWH without MetS and PLWH with MetS (**Supplementary Data File S2**, FDR <0.001), using Mann-Whitney U and limma, respectively. Moreover, PLS-DA was used to identify variations between the groups based on lipid concentrations, by exploiting its ability to handle a greater number of features compared to samples. The separation of the two groups was indicated by a score plot, where the two first orthogonal components explained half of the variance in the data with 45% and 5%, respectively. We found 516 lipids with VIP values > 1, Q2Y = 0.319 (**Supplementary Data File S2**). To obtain a better model performance we could increase the sample size. Finally, we created three types of RF models, minimal-optimal (‘Min’), geometric mean (‘Mid’) and all-relevant (‘Max’) models, which represented feature selection with minimal number of misclassifications, where we observed good performance of all models (**Figure 2A**, AUC>82.9%). Then, we identified 13 lipids as the strongest predictors of separating PLWH without MetS and PLWH with MetS (**Figure 2B**, ‘Max’ MUVR model, AUC = 83%), where the glycerolipid classes DAGs and TAGs were found to have the greatest significance in group separation. The number of significant lipids identified by each of the four methods varied greatly (**Supplementary Data File S2**). However, we observed 13 differentially abundant lipids between PLWH without MetS and PLWH with MetS (**Figure 2C**) which were consistently identified in all four methods (i.e., Mann-Whitney U, limma, PLS-DA, and RF). These 13 key lipids and 11 key metabolites (**Table 2**) indicated relatively good separation between PLWH without MetS and PLWH with MetS on sample clustering (**Figure 2D**). Furthermore, we observed higher abundance level of the key lipids in PLWH with MetS compared to PLWH without MetS (**Figure 2E**).

**Table 1:** Clinical and demographic characteristics compared between PLWH without MetS and PLWH with MetS.

Variables	PLWH without MetS	PLWH with MetS	pvalue
Sample (n)	100	100	
Sex, Male, n (%)	90 (90.0)	90 (90.0)	1.00**
Age, mean (sd)	54.4 (9.5)	54.6 (8.5)	0.80*
Ethnicity, n (%)			0.87**
Caucasian	88 (88.0)	86 (86.0)	
Asian	3 (3.0)	2 (2.0)	
Black	4 (4.0)	6 (6.0)	
Other/unknown	5 (5.0)	6 (6.0)	
Immunodeficiency, n (%)	14 (14.0)	13 (13.0)	1.00**
Exposure to early-generation ART, n (%)	34 (34.0)	46 (46.0)	0.11**
VAT, mean (sd)	76.1 (53.6)	149.4 (71)	< 0.001*
SAT, mean (sd)	111.1 (71.1)	150.6 (77.1)	< 0.001*
ART_NRTI, n (%)	95 (95.0)	96 (96.0)	1.00**
ART_NNRTI, n (%)	54 (54.0)	45 (45.0)	0.26**
ART_PI, n (%)	37 (37.0)	47 (47.0)	0.20**
ART_INSTI, n (%)	16 (16.0)	21 (21.0)	0.47**
ART_other/unknown, n (%)	0 (0.0)	3 (3.0)	0.24*

*Mann-Whitney U test and **Chi-square test.

P-values in bold indicates a significant difference in the concerned variables between the two groups. Immunodeficiency was defined as the lowest CD4+ T-cell count <200 cells/µl or previous AIDS condition and exposure to early-generation ART was defined as patients medicated with thymidine analogues, didanosine and/or indinavir.

**Table 2:** Identified key lipids and key metabolites.

Key lipids	Key metabolites
DAG(16:0/18:1)	1-carboxyethylisoleucine
DAG(16:0/18:3)	4-cholesten-3-one
TAG(44:0)-FA(18:0)	4-hydroxyglutamate
TAG(52:2)-FA(16:0)	*α*-ketoglutarate
TAG(52:2)-FA(18:1)	carotene diol (2)
TAG(52:2)-FA(18:2)	*γ*-glutamylglutamate
TAG(52:3)-FA(18:1)	glutamate
TAG(54:3)-FA(16:0)	glycerate
TAG(54:3)-FA(20:2)	isoleucine
TAG(54:3)-FA(20:3)	PC/3-MAPC*
TAG(54:4)-FA(16:0)	PSP**
TAG(54:4)-FA(20:3)	
TAG(54:5)-FA(16:0)	

*pimeloylcarnitine/3-methyladipoylcarnitine (C7-DC).

**palmitoyl-sphingosine-phosphoethanolamine (d18:1/16:0).

Overview of key lipids and key metabolites with significant differential abundance between PLWH without MetS and PLWH with MetS. Listed in alphabetical order.

### Structural Interpretation of Lipids Indicates Compositional Lipid Patterns

**Figure 3:**
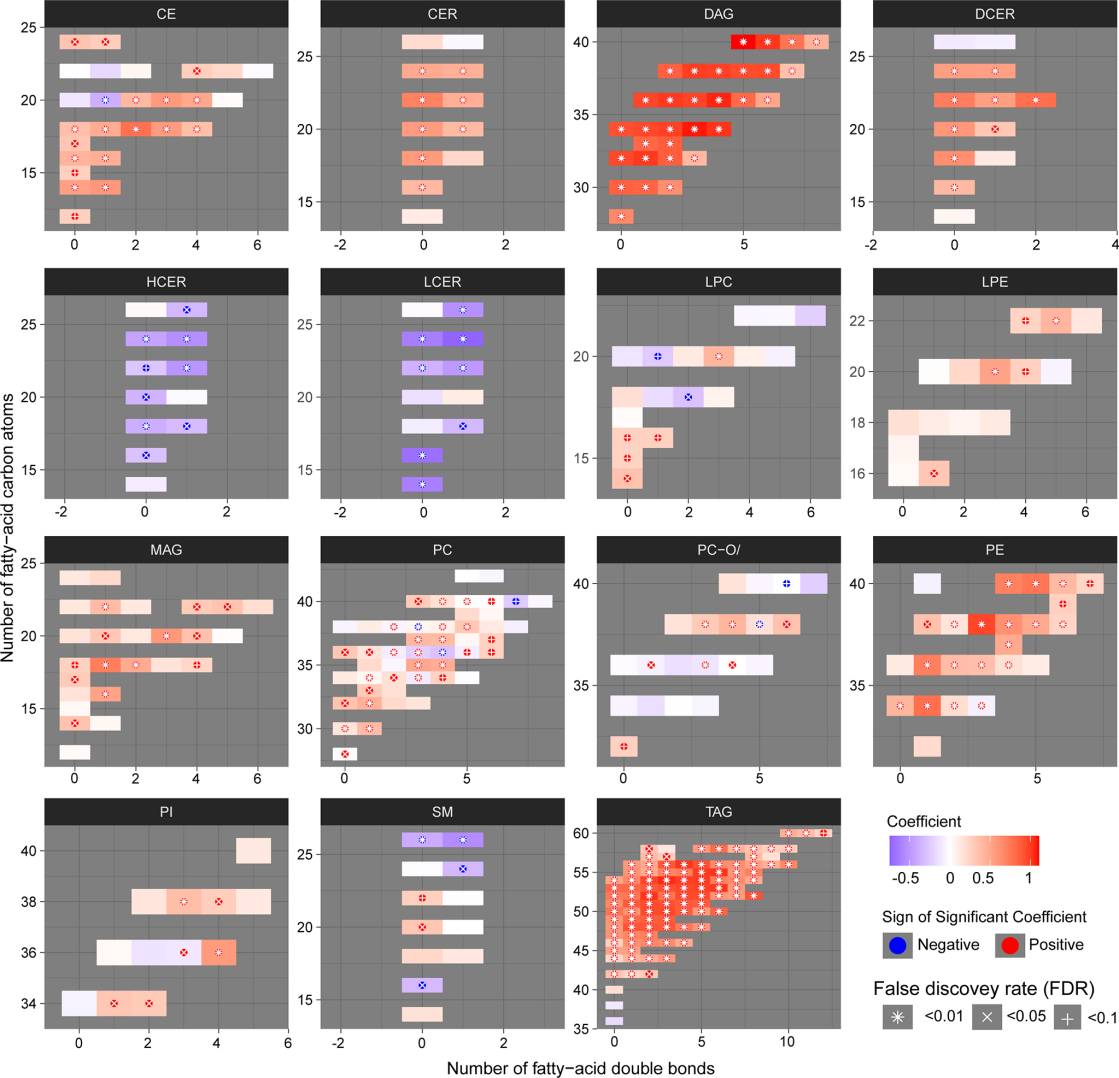
Structural differences of the lipidomic profile of PLWH without MetS vs. PLWH with MetS. Heatmaps for each lipid class show the structural lipid composition differences between PLWH without MetS and PLWH with MetS. Each lipid species is shown as a rectangle and the color shows the abundance difference (red: higher in PLWH with MetS; white: no difference; blue: lower in PLWH with MetS), the lipids were organized by the lipid size (y-axis) and level of saturation (x-axis). Lipids with statistically significant differences between the two groups were highlighted with a symbol. P-values have been FDR adjusted.

We then examined the structural characteristics of the lipidome by lipid class in terms of FA carbon number and saturation level (**Figure 3**). We observed an increase of ceramide (CER), DAG, dihydroceramide (DCER), lysophosphatidylethanolamine (LPE), monoacylglyceride (MAG), PE and TAG, in PLWH with MetS compared to PLWH without MetS, and a decrease in hexosylceramide (HCER) and lactosylceramide (LCER) (**Figure 3**, FDR<0.01 and Pearson’s r>0.7). An increased significantly differential abundance of DAGs and TAGs was observed, indicated by the symbol and red color. The TAGs tended to display a higher abundance of polyunsaturated lipids (i.e., a double-bond content between 2-5) with long-chain fatty acids (LCFA) (i.e., C48-56) (**Figure 3**, FDR<0.01, Pearson’s r>0.7). Additionally, TAGs displayed the most significant amount of lipid species. DAGs showed a tendency of increase in both saturated and unsaturated lipids (i.e., a double-bond content between 0-6) with LCFA (i.e., C30-40) (**Figure 3**, FDR<0.01, Pearson’s r>0.7).

### Clinical and Omics Integrated Network Identifies Biomolecular Patterns

Seeking to test whether and how any coordinated patterns of association were present throughout the samples, we generated weighted lipid-metabolite networks to understand the relationship between lipidomic profiles with those metabolites associated with MetS in PLWH. While retaining only informative metabolites, we examined the relationship between key metabolites previously identified with the entire lipidome. Briefly, we associated clinical variables with the identified communities and within the most central community, we identified associations between clinical variables and each biomolecule.

**Figure 4:**
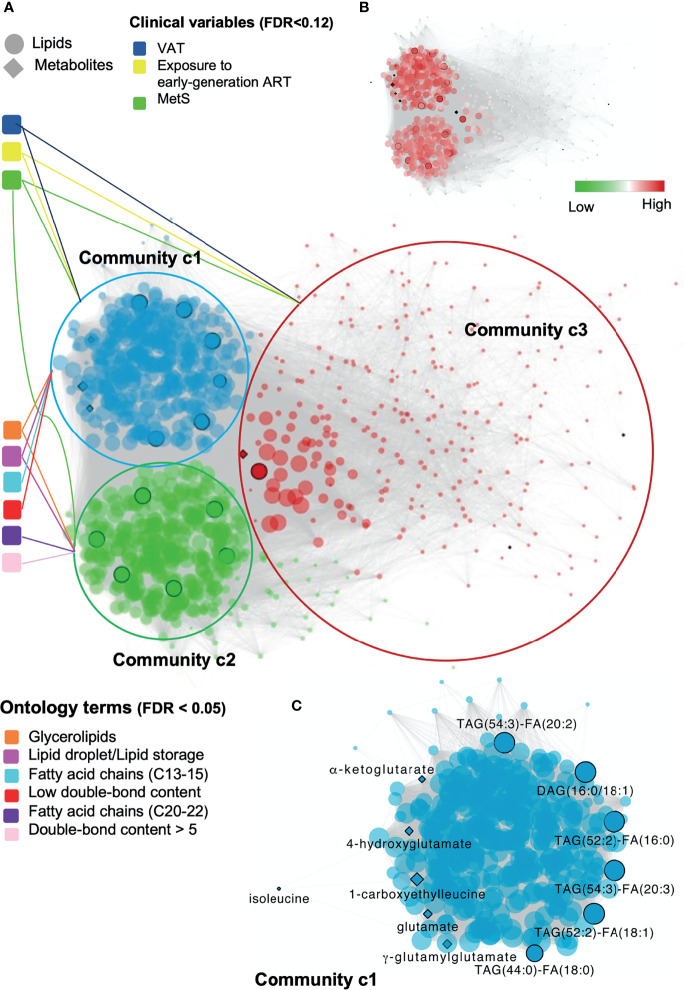
Global and local biomolecular network of PLWH without MetS vs PLWH with MetS. **(A)** Global network illustrating the associated clinical variables and ontology terms with each community. Network of positive correlations between lipids and metabolites (FDR < 1e-07, Spearman’s ρ > 0.38), colored based on the three identified communities, c1 (blue), c2 (green) and c3 (red). Communities are connected with associated clinical variables (FDR < 0.12) and ontology terms (FDR < 0.05). Black circled lipids and metabolites correspond to identified key lipids and key metabolites (**Table 2**). **(B)** Global network illustrating up and down-regulated lipids in PLWH with HIV. **(C)** Local network of community c1 highlighting key biomolecules. Biomolecular correlations within community c1 (FDR < 1e-07, Spearman’s ρ > 0.38). Black circled and named biomolecules correspond to the identified key lipids and metabolites within c1.

The fully connected biological network comprised 18430 edges and 917 nodes and displayed markedly distinct behavior from the null network (**Supplementary Table S1** and **Supplemetary Figure S1**). A community analysis on the biological network identified three communities of strongly interconnected lipids and metabolites (**Supplementary Table S2**). Centrality properties were evaluated identifying c1 as the most central community in the network (**Figure 4A**), which captured the most coordinated differential abundance changes. Community c1 had the largest community size (size = 339) and largest community average degree (avg. degree = 534.63) (**Supplementary Table S2**).

Structural and functional characterization of these communities (23) (**Supplementary Figure S2**) indicated that glycerolipids and especially TAGs were enriched in both c1 and c2 (**Figure 4A**, FDR<0.05). Interestingly, a coordinated structural composition pattern of the glycerolipids displayed an average lower carbon number and average lower double-bond content in c1, compared to c2 (**Supplementary Table S3**). Community c3 was not further addressed, as the two other communities were interpreted to be of more importance due to their node size and average degree (**Supplementary Table S2**). We identified a positive association between community c1 with the clinical variables MetS, VAT and exposure to early-generation ART (**Supplementary Table S4**, FDR<0.12, illustrated in **Figure 4A**). In turn, community c2 was positively associated with MetS, however with a lower estimate compared to c1 (**Supplementary Table S4**, FDR<0.12). Log-fold changes indicated up-regulation of lipids in PLWH with MetS compared to PLWH in communities c1 and c2 (**Figure 4B**, limma, FDR<0.001).

Furthermore, we observed a positive association between lipids (i.e., DAGs and TAGs) and VAT (**Supplementary Table S5**, FDR<0.01). TAGs tended to consist of polyunsaturated lipids (i.e., a double-bond content ≥2) with LCFA (i.e., C48-54). Interestingly, we also observed that TAGs with LCFA (i.e., C42-48) and a low double-bond content (i.e., ≤2) were positively associated with the use of NNRTI (**Supplementary Table S5**, FDR<0.07). Additionally, four out of the 13 key lipids [i.e., TAG(52:2)-FA(16:0), TAG(52:2)-FA(18:1), DAG(16:0/18:1), and TAG(54:3)-FA(20:3)] were all found to be independently associated with VAT (**Supplementary Table S5**, FDR<0.01). Finally, one out of the 11 key metabolites (i.e., glutamate) was also found to be independently associated with VAT (**Supplementary Table S5**, FDR<0.01).

The top 10% of most interconnected biomolecules, found according to their degree, were all glycerolipids within the classes DAG and TAG (**Supplementary Table S6**). Three out of the 13 key lipids [i.e., TAG(52:2)-FA(16:0), TAG(52:2)-FA(18:1) and TAG(54:3)-FA(20:3)] were ranked among the top 10% most interconnected biomolecules in c1. Thus, these three lipids were interpreted to be among those biomolecules influencing the behavior of the global network the most. It should be noticed that the structural composition of all three lipids are polyunsaturated TAGs with LCFA and were all found to be positively associated with VAT (**Supplementary Table S5**, FDR<0.01).

The local network of community c1 included six out of the 13 key lipids (i.e., TAG(54:3)-FA(20:2), DAG(16:0/18:1), TAG(52:2)-FA(16:0), TAG(54:3)-FA(20:3), TAG(52:2)-FA(18:1), and TAG(44:0)-FA(18:0), **Figure 4C**). All of the six key lipids within c1 were glycerolipids, five TAGs and one DAG. Interestingly, we observed that all five TAGs were polyunsaturated with a doubsle-bond content between 2 and 4 with a FA carbon number between C52 and C54. Moreover, the key lipids were found to be interconnected with 6 of the 11 key metabolites within c1. Finally, all 13 key lipids and 7 metabolites within the global network were found to be interconnected with each other.

## Discussion

The integrative plasma lipidomics and metabolomics analysis in a large HIV cohort of PLWH with and without MetS indicated a complex role of lipids in the link between ART and MetS in PLWH and provided a system-level understanding of MetS in PLWH. Our data indicated an increased abundance of the glycerolipids DAGs and TAGs in PLWH with MetS. The comprehensive network integration of the lipidomics and metabolomics (8) data suggested interactions between specific glycerolipids patterns and key metabolites involved in the glutamate metabolism. Finally, our data also indicated a relationship between the structural composition patterns of these specific glycerolipids with HIV and MetS-specific clinical variables, suggesting their involvement in driving the disease pathogenesis in PLWH with MetS.

In our study, we found 13 key glycerolipids from the classes DAG (n = 2) and TAG (n = 11) to be significantly altered between PLWH with and without MetS. It is worth noting the structural composition of the 13 lipids. The two DAGs [DAG(16:0/18:1) and DAG(16:0/18:3)] consist of unsaturated LCFA (i.e., C34 and 1-3 double-bonds). Additionally, 10 out of the 11 TAGs were polyunsaturated LCFA (i.e., C52-54 and a double-bond content of 2-5). The last TAG had a lower carbon number of C44, compared to the others and was saturated. These findings support previous findings of MetS in general populations that showed that lipids (especially TAGs) with lower carbon numbers (i.e., C44-54) and lower double-bond content (i.e., 1-4) were associated with an increased risk of T2D. Moreover, it had been observed that an increase in DAGs was associated with hypertension, another MetS-related factor (24). The structure of the FAs is a useful indication of the functionality of lipid metabolism. Increased accumulation of LCFA such as C(16:0), C(16:1), C(18:0), and C(18:1) suggests increased biosynthesis under MetS-conditions. Such chain compositions are observed among our 13 identified key lipids both in the DAGs and TAGs. Additionally, to the observed pattern of LCFAs, another study suggests LCFAs might cause impairment of mitochondria functions (25).

We employed network analysis by integrating the key metabolites previously identified (8) as biomarkers in PLWH with MetS and the lipids with the clinical features (phenomics) that can provide a comprehensive view of the metabolic state related to a disease phenotype. Interestingly, we observed that community c1 contained glycerolipids with a lower carbon number and lower double-bond content compared to c2. Community c1 was further investigated and we found that c1 positively associated with the clinical variables MetS, VAT, and exposure to early-generation ART. Our findings are related to previous findings that showed TAGs with a lower carbon number and lower double-bond content play a considerable role in MetS (23, 26). Additionally, our results suggest that exposure to early-generation ART (i.e., thymidine analogues/didanosine/indinavir) and increased VAT may also lead to a lower carbon number and double-bond content in glycerolipids, suggesting a role for polyunsaturated glycerolipids with LCFA [i.e., especially TAG(52:2)-FA(16:0), TAG(52:2)-FA(18:1) and TAG(54:3)-FA(20:3)] in the metabolic patterns in PLWH with MetS.

Previous studies have investigated the association of lipidomics profiles (211 lipids) with the progression of CVD in PLWH receiving ART treatment with carotid artery atherosclerosis, compared to HIV-negative individuals (3). The study showed elevation in lipid species with polyunsaturated LCFAs (i.e., C13-21 and double-bond content ≥2) in patients with atherosclerosis, which is also observed in PLWH with MetS in our study. Additionally, their study suggested significant alterations in lipid species such as CE, CER, LPC, LPE, PC, PIs, and PI. Other studies in both HIV and HIV-negative populations also found alterations in levels of other lipid species (i.e., different from DAG and TAG) to be associated with MetS-factors. This includes CE, CER, LPC, PC, PE, and sphingomyelin (SM) (5, 23). Some of these lipid species were also altered in our study population (i.e., CE, CER, PE, and SM); however the glycerolipids showed strongest predictive values. Our findings of coordinated abundance shift in glycerolipids may be due to our considerably larger amount of quantified lipid species than other studies (3, 23, 26).

In the same cluster (i.e., c1) we observed two trends concerning coordinated abundance shifts in glycerolipids. First, TAG species with carbon numbers between C48-54 and double-bond content ≥2, together with DAG species with carbon number between C32-36 and double-bond content ≥1 associated positively with VAT (FDR<0.01). This finding correlates with previous studies of MetS factors in HIV-negative cohorts (23, 26). Second, TAG species with carbon numbers between C42-48 and double-bond content ≤2 positively associated with the use of ART drugs containing NNRTIs (FDR<0.07). The latter trend supports previous findings suggesting that the NNRTIs drug efavirenz introduces dysfunction in the mitochondria by inducing increased levels of lipids (27). Moreover, both exposures to early-generation ART and the use of NNRTIs drugs have shown to cause disruption of the mitochondrial functions in previous studies (27, 28). To our knowledge, we present here the first evidence of association of antiretroviral treatment with specific structural composition lipid profiles.

The composition-specific glycerolipids correlated with some of the previously identified key metabolites linked to the perturbations of the glutamate metabolism in PLWH with MetS (8). This finding correlates with previous studies of MetS in HIV-negative populations, which found branched-chain amino acids (BCAAs) (i.e., leucine, isoleucine, and valine) as one of the significant metabolite groups dysregulated in obese individuals together with increased concentrations of glutamate, which is the first step of the BCAAs catabolism (29, 30). Additionally, the polar metabolite acylcarnitine (abbreviated PC/3-MAPC), an essential member of the fatty acid metabolism, was significantly down-regulated in PLWH with MetS. This molecule facilitates the transportation of LCFAs into the mitochondria for catabolism through *β*-oxidation (31). Besides glutamate and 4-hydroxyglutamate being a part of the glutamate metabolism, other identified key metabolites were a part of mitochondrial processes, which have critical energetic functions (e.g., regulating insulin secretion). These metabolites belonged to the isoleucine metabolism (i.e., 1-carboxyethylleucine, isoleucine) and the TCA cycle (i.e., *γ*-glutamylglutamate, *α*-ketoglutarate) *(* 8).

The present study has limitations, such as the cross-sectional design, as no conclusions on causality could be drawn. We were only able to assess the prevalence of the diseases in the plasma samples. Finally, despite the largest study population conducted to date to type comprehensive lipid profile in PLWH, the relatively small sample size of the cohort is also considered as a limitation to this study. However, this is the first study that includes a large, well-characterized group of PLWH with or without MetS matched on MetS, sex, and age. Furthermore, the use of fully quantitative lipidomics (i.e., >900 quantified lipid species) methodology allowed us to conduct a thorough analysis of the systematic lipid profiling and its association with metabolites and clinical factors by using a combination of standard biostatistical, machine learning, and network analysis techniques.

## Conclusions

In conclusion, our study suggests alterations in both fatty acid metabolism and glutamate metabolism, which depend on well-functioning mitochondria. A synergistic effect of different factors (i.e., an increased pro-inflammatory state induced by HIV, age-related pathophysiological changes, exposure to early-generation ART, and the use of ART with the active agent NNRTIs), which perturb the functions within the biological system of HIV-infected, could play a part in the alterations of the identified biological mechanisms in the phenotype PLWH with MetS. A recent large study from India and Cameroon also reported alterations in glutaminolysis as a common factor in PLWH in long-term cART (32). Alterations in the lipid homeostasis, as well as glutaminolysis in PLWH, need clinical or dietary interventions as they might drive accelerated aging in PLWH with MetS. A recent report indicated that the senescent cells depend on the glutaminolysis, and inhibition of the pathway leads to both inducing senolysis (i.e., removal of senescent cells) as well as improved serum-free fatty acids (FFAs) in the aged mice, which is a hallmark of the obesity-related disorders. We, therefore, hypothesized that clearance of the senescent cells by inhibition of the glutaminolysis and improving the lipid profile might prevent age-associated disorders and provide healthy aging in the PLWH with MetS. This further can aid in developing therapeutic targets to avoid metabolic abnormalities and accelerated aging in PLWH with MetS.

## Data Availability Statement

The datasets presented in this study can be found in online repositories. The names of the repository/repositories and accession number(s) can be found in the article/**Supplementary Material**.

## Ethics Statement

The studies involving human participants were reviewed and approved by Regional Ethics Committee of Copenhagen (COCOMO: H-15017350). The patients/participants provided their written informed consent to participate in this study.

## Author Contributions

Conceptualization and clinical study designing: SN, SO, UN, MG, AK, and DM. Clinical data and biobank: SN, MG, AK, JH, MT, and HU. Methodology: SO, RB, and UN. Formal analysis: SO and RB. Clinical interpretation: SO, UN, RB, SN, MG, AK, and DM. Supervision: RB, AK, MG, UN, and SN. Resources: UN and SN. Writing (original draft): SO. Writing (review and editing): RB, AK, MG, HU, MT, JH, DM, UN, and SN. Visualization: SO, RB, and UN. Project administration: UN and SN. Funding acquisition: UN and SN. All authors discussed the results, commented, and approved the final version of the manuscript.

## Funding

The study is funded by Rigshospitalet Research Council, Danish National Research Foundation (DNRF126) NovoNordisk Foundation. UN acknowledges the support received from Swedish Research Council Grants (2017-01330, 2018-06156, and 2021-01756).

## Conflict of Interest

The authors declare that the research was conducted in the absence of any commercial or financial relationships that could be construed as a potential conflict of interest.

## Publisher’s Note

All claims expressed in this article are solely those of the authors and do not necessarily represent those of their affiliated organizations, or those of the publisher, the editors and the reviewers. Any product that may be evaluated in this article, or claim that may be made by its manufacturer, is not guaranteed or endorsed by the publisher.
