# Supplementary material for: Integrative Lipidomics and Metabolomics for System-Level Understanding of the Metabolic Syndrome in Long-Term Treated HIV-Infected Individuals
Source: Front Immunol. 2022 Jan 12;12:742736. doi: 10.3389/fimmu.2021.742736 (PMC8791652; doi:10.3389/fimmu.2021.742736)
Supplement: Supplementary file 1 [file DataSheet_1.pdf]

Supplementary Material

1 Supplementary Data

Supplementary Data File S1: Original Scale Lipidomics Data.

Supplementary Data File S2: Overview of statistical outcomes from Mann Whitney U test, limma, PLS-DA and random forest. Outcome from statistical and machine learning methods identifying lipid species differentiating PLWH without MetS from PLWH with MetS. The table includes significant lipid species and their super pathway together with results from the four used methods Mann Whitney u test (pvalue and FDR adjusted pvalues), limma (pvalue and FDR adjusted pvalues), PLS-DA (variable importance on projection (VIP) values) and random forest (lowest rank indicating strongest predictors of separating PLWH without MetS from PLWH with MetS). Cells with hyphens illustrates that the concerned lipid specie is found not to significantly differentiating the two groups by the concerned method.

The lipidomics data can be obtained from the dx.doi.org/10.6084/m9.figshare.14509452

2 Supplementary Figures and Tables

2.1 Supplementary Figures

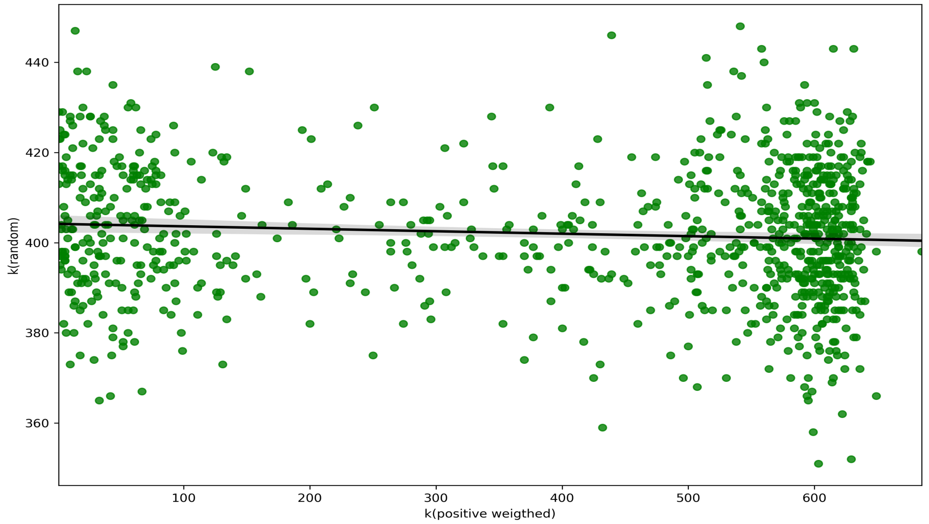

Supplementary Figure S1: Degree distribution for the positive weighted against the random network.

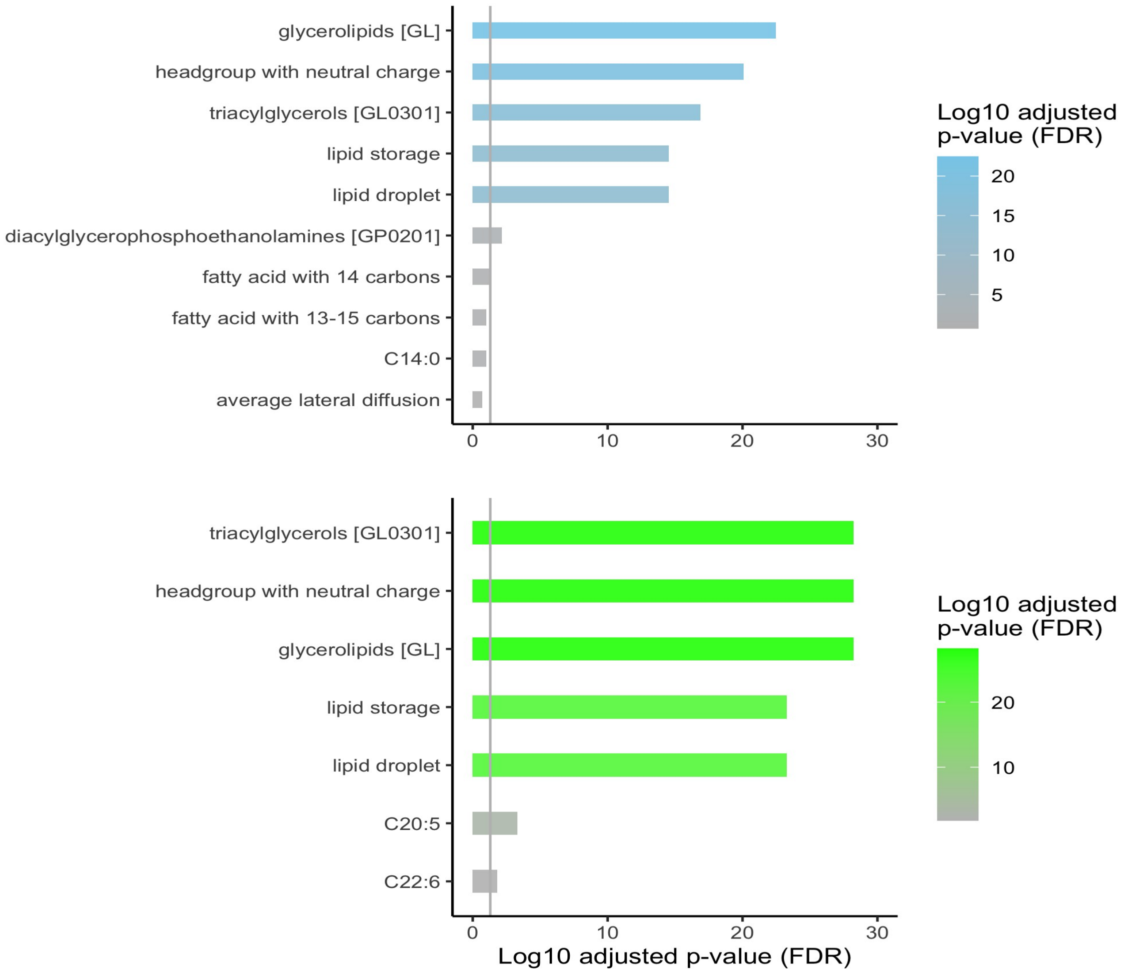
glycerolipids [GL]
headgroup with neutral charge
triacylglycerols [GL0301]
lipid storage
lipid droplet
diacylglycerophosphoethanolamines [GP0201]
fatty acid with 14 carbons
fatty acid with 13-15 carbons
C14:0
average lateral diffusion
0
10
20
30
Log10 adjusted p-value (FDR)
20
15
10
5
triacylglycerols [GL0301]
headgroup with neutral charge
glycerolipids [GL]
lipid storage
lipid droplet
C20:5
C22:6
0
10
20
30
Log10 adjusted p-value (FDR)
Log10 adjusted p-value (FDR)
20
10

a.

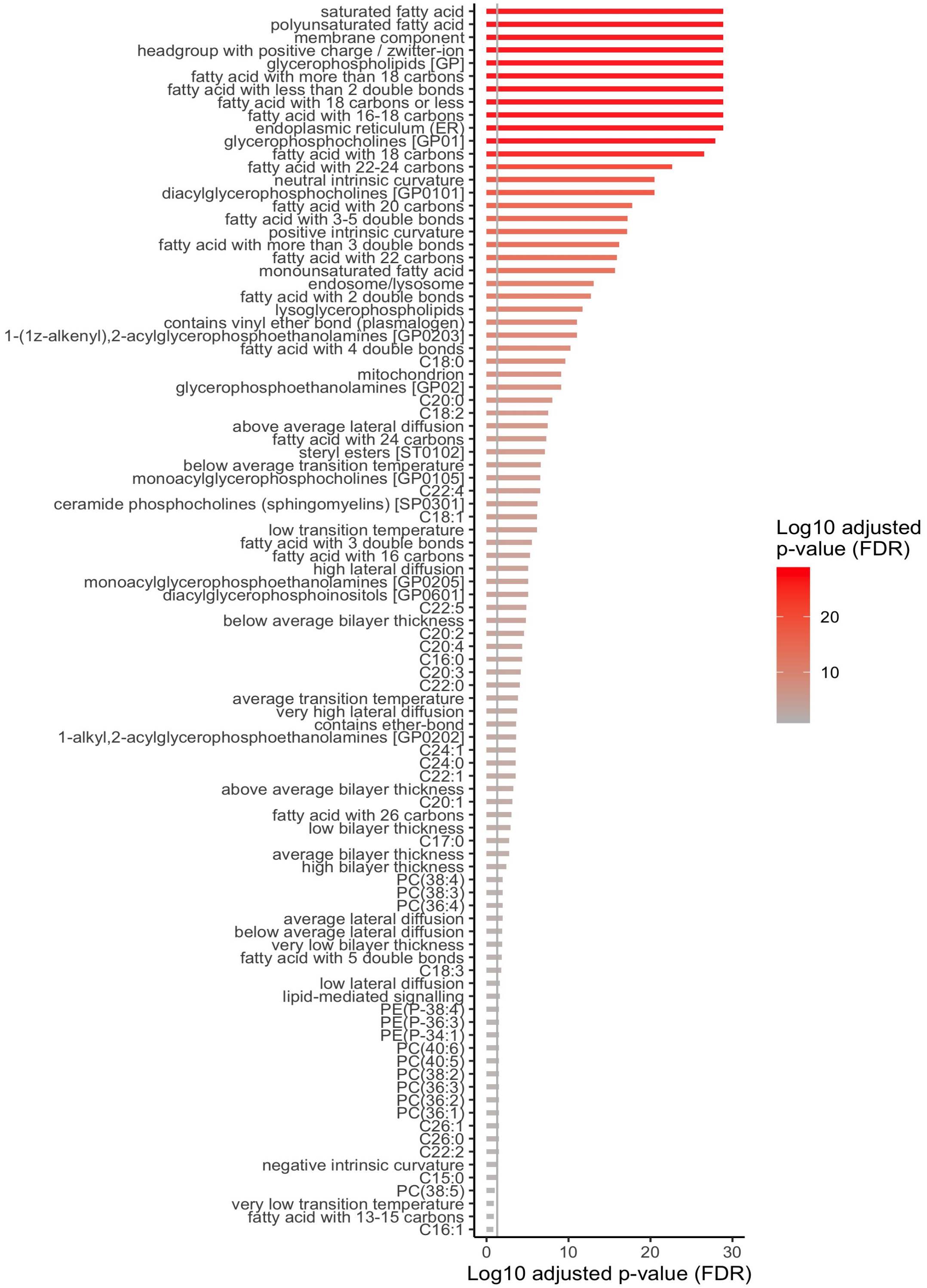

b.

Supplementary Figure S2: Ontology enrichment plots for the three communities c1, c2 and c3. (a) Ontology enrichment plots for community c1 (blue) and c2 (green). (b) Ontology enrichment plot for community c3.

2.2. Supplementary Tables

Supplementary Table S1: Network properties of the positive and random network. Including type of network (Network), node count (Nodes), edge count (Edges), average degree of network (AvgD), average path length (AvgPL), clustering coefficient (CC), if the network is connected or not (C?) and the minimum cut off (MinCut).

Network	Nodes	Edges	AvgD	AvgPL	CC	C?	MinCut
Positive weighted	917	184304	402	1.72	0.87	True	1
Random	917	184304	402	1.56	0.44	True	351

Supplementary Table S2: Table of community properties including size and average degree of the three identified communities. The size corresponds to the number of lipids and metabolites (nodes) within each community. The average degree of the community corresponds to how connected lipids and metabolites (nodes) within the concerned community are.

Community	Biomolecules	Avg. degree	Lipids	Metabolites
c1	339	534.63	333	6
c2	318	506.58	318	0
c3	260	101.05	257	3

Supplementary Table S3: A structural composition table, providing an overview of composition of enriched glycerolipids in the network communities c1 and c2.

Communities	c1	c2
Biomolecules in community	339	264
TAGs in community (n, %)	252 (74%)	318 (83%)
Average TAG carbon number	49 (38-56)	55 (48-60)
Average TAG double-bond content	2 (0-8)	5 (0-12)
DAGs in community (n, %)	28 (8%)	24 (9%)
Average DAG carbon number	32.5 (28-36)	37 (34-40)
Average DAG double-bond content	1.5 (0-5)	5 (2-8)

Supplementary Table S4: Correlation table predicting clinical variables based on the community score (FDR < 0.12). Ranked according to FDR (p-value adj).

Community	Clinical variable	estimate	std. error	t value	p-value	p-value adj
c1	MetS	0.29	0.04	7.31	6.48e-12	< 0.000001
c3	MetS	0.31	0.04	7.30	6.73e-12	< 0.000001
c1	VAT	36.34	6.07	5.99	9.83e-09	< 0.000001
c3	VAT	42.96	6.15	6.98	4.31e-11	< 0.000001
c3	Exposure preART	0.13	0.05	2.86	0.004	< 0.12
c2	MetS	0.19	0.08	2.32	0.019	< 0.12
c1	Exposure preART	0.10	0.04	2.36	0.019	< 0.12

Supplementary Table S5: Association table predicting clinical variables based on each lipid and metabolite concentration (FDR < 0.07) within community c1. The model is adjusted for MetS, sex and age. Ranked according to FDR (p-value adj). Five key lipids and one key metabolite were associated with VAT and marked in bold in the table.

Clinical variable	Biochemicals	estimate	std. error	t value	p-value	p-value adj
ART_PI	DAG(14:0/18:3)	0.25	0.03	8.02	9.51e-14	< 1 e-6
VAT	TAG(52:2)-FA(16:0)	22.86	4.72	4.85	3,00E-06	< 0.01
VAT	TAG(52:2)-FA(18:1)	22.43	4.75	4.72	4,00E-06	< 0.01
VAT	glutamate	20.04	4.44	4.51	1.1e-5	< 0.01
VAT	TAG(50:3)-FA(16:0)	21.39	4.75	4.5	1.2e-5	< 0.01
VAT	TAG(54:4)-FA(20:4)	21.04	4.63	4.54	1,00E-05	< 0.01
VAT	DAG(18:0/18:1)	20.45	4.58	4.47	1.3e-5	< 0.01
VAT	TAG(56:4)-FA(22:4)	20.59	4.62	4.45	1.4e-5	< 0.01
VAT	TAG(56:5)-FA(22:4)	20.6	4.62	4.46	1.4e-5	< 0.01
VAT	TAG(54:5)-FA(22:5)	20.03	4.62	4.33	2.3e-5	< 0.01
VAT	DAG(16:0/18:1)	20.44	4.75	4.31	2.6e-5	< 0.01
VAT	TAG(50:4)-FA(16:0)	20.05	4.72	4.25	3.3e-5	< 0.01
VAT	TAG(50:2)-FA(16:0)	20.09	4.78	4.2	4,00E-05	< 0.01
VAT	DAG(16:0/20:4)	19.64	4.7	4.18	4.4e-5	< 0.01
VAT	TAG(51:3)-FA(16:0)	19.45	4.72	4.12	5.6e-5	< 0.01
VAT	TAG(52:1)-FA(18:1)	19.04	4.62	4.12	5.6e-5	< 0.01
VAT	TAG(50:2)-FA(18:2)	19.11	4.68	4.08	6.6e-5	< 0.01
VAT	TAG(50:3)-FA(18:3)	19.05	4.67	4.08	6.4e-5	< 0.01
VAT	TAG(51:4)-FA(16:0)	19.34	4.73	4.08	6.4e-5	< 0.01
VAT	TAG(54:3)-FA(20:3)	18.91	4.71	4.01	8.6e-5	< 0.01
VAT	TAG(50:1)-FA(16:0)	18.68	4.72	3.96	1.06e-4	< 0.07
VAT	TAG(52:1)-FA(16:0)	18.23	4.6	3.96	1.06e-4	< 0.07
VAT	TAG(50:3)-FA(18:2)	18.86	4.79	3.94	1.13e-4	< 0.07
VAT	TAG(54:4)-FA(22:4)	18.09	4.63	3.9	1.3e-4	< 0.07
VAT	TAG(50:1)-FA(18:1)	17.92	4.72	3.79	1.98e-4	< 0.07
VAT	TAG(52:2)-FA(18:0)	17.43	4.59	3.8	1.95e-4	< 0.07
ART_PI	PI(16:0/20:3)	0.13	0.03	3.74	2.43e-4	< 0.07
VAT	TAG(53:4)-FA(16:0)	17.31	4.68	3.7	2.83e-4	< 0.07
VAT	TAG(54:3)-FA(20:2)	17.4	4.74	3.67	3.13e-4	< 0.07
VAT	TAG(52:3)-FA(20:3)	17.13	4.72	3.63	3.64e-4	< 0.07
VAT	TAG(52:4)-FA(20:4)	16.51	4.6	3.59	4.21e-4	< 0.07
VAT	TAG(51:3)-FA(18:3)	16.34	4.61	3.54	4.95e-4	< 0.07
VAT	TAG(50:2)-FA(16:1)	16.84	4.76	3.54	5,00E-04	< 0.07
VAT	DAG(16:1/20:4)	16.53	4.67	3.54	5.09e-4	< 0.07

VAT	TAG(52:1)-FA(18:0)	15.98	4.55	3.52	5.46e-4	< 0.07
VAT	DAG(16:1/18:0)	16.17	4.63	3.49	5.91e-4	< 0.07
VAT	TAG(51:2)-FA(16:0)	16.44	4.73	3.47	6.32e-4	< 0.07
VAT	TAG(50:2)-FA(18:1)	16.55	4.79	3.45	6.79e-4	< 0.07
VAT	TAG(53:3)-FA(16:0)	15.82	4.63	3.42	7.75e-4	< 0.07
VAT	DAG(16:0/16:1)	15.97	4.73	3.37	8.98e-4	< 0.07
VAT	TAG(51:3)-FA(17:0)	15.88	4.71	3.37	8.98e-4	< 0.07
VAT	TAG(53:2)-FA(17:0)	15.54	4.6	3.37	8.91e-4	< 0.07
VAT	TAG(54:5)-FA(22:4)	15.92	4.72	3.37	8.99e-4	< 0.07
VAT	TAG(52:5)-FA(20:4)	15.7	4.67	3.36	9.46e-4	< 0.07
VAT	TAG(50:1)-FA(16:1)	15.49	4.65	3.33	0.001044	< 0.07
SAT	TAG(48:4)-FA(18:1)	-16.1	4.9	-3.29	0.001196	< 0.07
VAT	TAG(54:2)-FA(20:2)	15.33	4.66	3.29	0.0012	< 0.07
ART_NNRTI	TAG(48:2)-FA(12:0)	0.12	0.04	3.28	0.00123	< 0.07
VAT	TAG(53:4)-FA(20:4)	15.17	4.63	3.28	0.001238	< 0.07
VAT	DAG(16:0/18:0)	14.99	4.58	3.27	0.001258	< 0.07
VAT	TAG(52:4)-FA(22:4)	15.11	4.62	3.27	0.00126	< 0.07
VAT	TAG(51:2)-FA(18:2)	15.04	4.61	3.26	0.001304	< 0.07
VAT	TAG(53:5)-FA(20:4)	15.18	4.68	3.24	0.001385	< 0.07
ART_NNRTI	TAG(44:2)-FA(18:1)	0.12	0.04	3.23	0.001444	< 0.07
VAT	DAG(16:1/18:1)	15.36	4.75	3.23	0.001448	< 0.07
VAT	DAG(16:0/20:3)	15.36	4.76	3.23	0.001456	< 0.07
VAT	TAG(52:6)-FA(20:4)	15.19	4.71	3.23	0.001475	< 0.07
VAT	DAG(14:0/18:1)	14.97	4.66	3.21	0.001551	< 0.07
VAT	TAG(48:3)-FA(16:0)	14.89	4.64	3.21	0.001563	< 0.07
VAT	TAG(50:3)-FA(16:1)	15.1	4.75	3.18	0.00171	< 0.07
VAT	TAG(50:2)-FA(14:0)	14.77	4.66	3.17	0.001786	< 0.07
ART_NNRTI	CER(14:0)	0.11	0.04	3.16	0.001843	< 0.07
ART_NNRTI	TAG(42:1)-FA(18:1)	0.11	0.03	3.15	0.001879	< 0.07
VAT	TAG(48:2)-FA(18:2)	14.38	4.57	3.15	0.001896	< 0.07
VAT	TAG(51:1)-FA(17:0)	14.49	4.6	3.15	0.001913	< 0.07
VAT	TAG(48:3)-FA(14:0)	14.73	4.69	3.14	0.001945	< 0.07
VAT	TAG(50:0)-FA(16:0)	14.3	4.55	3.14	0.001937	< 0.07
VAT	TAG(52:2)-FA(16:1)	14.41	4.6	3.13	0.002008	< 0.07
VAT	TAG(51:2)-FA(15:0)	14.51	4.64	3.13	0.00203	< 0.07
ART_NNRTI	TAG(44:1)-FA(18:1)	0.11	0.04	3.12	0.002072	< 0.07

Supplementary Table S6: Top 10% nodes in community c1 based on the degree. Thus, the most interconnected nodes in the community. The lipids TAG(52:2)-FA(16:0), TAG(52:2)-FA(18:1) and TAG(54:3)-FA(20:3) are marked in bold, as they were among the key lipids.

Rank	Biochemicals	Degree
1	TAG(50:3)-FA(18:2)	639
2	TAG(51:3)-FA(17:0)	637
3	DAG(18:0/18:1)	636
4	TAG(50:3)-FA(16:0)	636
5	TAG(50:4)-FA(16:0)	636
6	TAG(53:2)-FA(17:0)	635
7	TAG(51:3)-FA(16:0)	634
8	TAG(53:4)-FA(16:0)	633
9	TAG(52:2)-FA(20:0)	632
10	TAG(51:3)-FA(16:1)	631
11	TAG(48:4)-FA(16:1)	630
12	TAG(50:2)-FA(14:0)	630
**13**	**TAG(52:2)-FA(16:0)**	**630**
14	TAG(52:2)-FA(16:1)	630
**15**	**TAG(52:2)-FA(18:1)**	**630**
16	TAG(50:3)-FA(18:1)	629
17	TAG(51:4)-FA(16:0)	629
18	TAG(52:4)-FA(14:0)	629
19	TAG(53:2)-FA(18:1)	629
20	TAG(54:5)-FA(22:5)	629
21	DAG(14:0/18:2)	628
22	TAG(51:2)-FA(18:1)	628
23	TAG(52:5)-FA(14:0)	628
24	TAG(54:4)-FA(20:4)	628
25	TAG(52:3)-FA(20:2)	627
26	TAG(50:2)-FA(18:1)	627
27	TAG(52:4)-FA(20:2)	627
28	TAG(52:5)-FA(20:3)	627
29	TAG(50:3)-FA(14:1)	626
30	TAG(53:5)-FA(20:4)	626
31	TAG(52:6)-FA(20:4)	625
**32**	**TAG(54:3)-FA(20:3)**	**625**
33	DAG(16:0/20:4)	624
34	TAG(52:7)-FA(18:1)	624
